# Supplementary material for: Alterations in gut microbiota linked to provenance, sex, and chronic wasting disease in white-tailed deer (Odocoileus virginianus)
Source: Sci Rep. 2021 Jun 24;11:13218. doi: 10.1038/s41598-021-89896-9 (PMC8225879; doi:10.1038/s41598-021-89896-9)
Supplement: Supplementary file 1 — Supplementary Information 1. [file 41598_2021_89896_MOESM1_ESM.docx]

**Supplementary Information**

**Alterations in gut microbiota linked to provenance, sex, and chronic wasting disease in white-tailed deer (*Odocoileus virginianus*)**

David Minich^1^, Christopher Madden^2^, Morgan V. Evans^2,3^, Gregory A. Ballash^1^, Daniel J. Barr^4^, Keith P. Poulsen^4^, Patricia M. Dennis^2,5^, Vanessa L. Hale^2*^

^1^The Ohio State University College of Veterinary Medicine, Columbus, OH

^2^Veterinary Preventive Medicine, The Ohio State University College of Veterinary Medicine, Columbus, OH

^3^Environmental Health Sciences, The Ohio State University College of Public Health

^4^Wisconsin Veterinary Diagnostic Laboratory, University of Wisconsin-Madison, WI

^5^Cleveland Metroparks Zoo, Cleveland, OH

*Corresponding author:

Vanessa L. Hale, MAT, DVM, PD

Department of Veterinary Preventive Medicine

Ohio State University College of Veterinary Medicine

1900 Coffey Rd.

Columbus, OH 43210

[hale.502@osu.edu](mailto:hale.502@osu.edu)

**Supplemental Figure 1 – Microbial composition by provenance and sex**:

Microbial composition (weighted UniFrac) differed significantly by provenance (PERMANOVA p = 0.001). Farm 1 deer are featured in green circles and red diamonds. Farm 2 deer are featured in blue circles and yellow diamonds. Free-ranging deer are featured in purple circles.


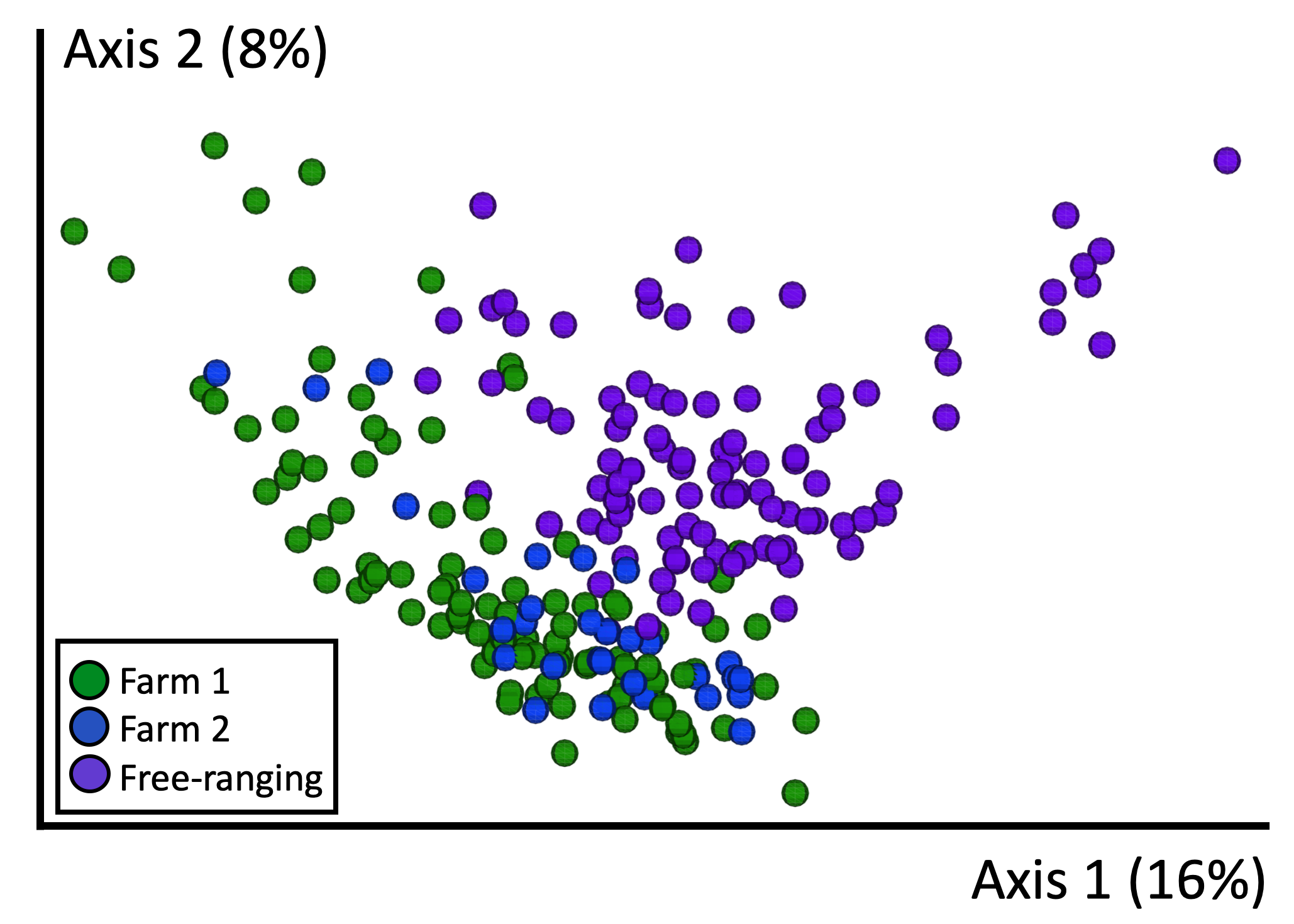


**Supplemental Figure 2 – Microbial composition by sex**

Gut microbial composition (unweighted UniFrac) by sex on a) Farm 1, b) Farm 2, and c) Free-ranging. Gut microbiota differed significantly by sex on Farm 1 (PERMANOVA p = 0.003), trended toward significance in free-ranging deer (PERMANOVA p = 0.066), but did not differ significantly on Farm 2 (PERMANOVA p = 0.115).

**
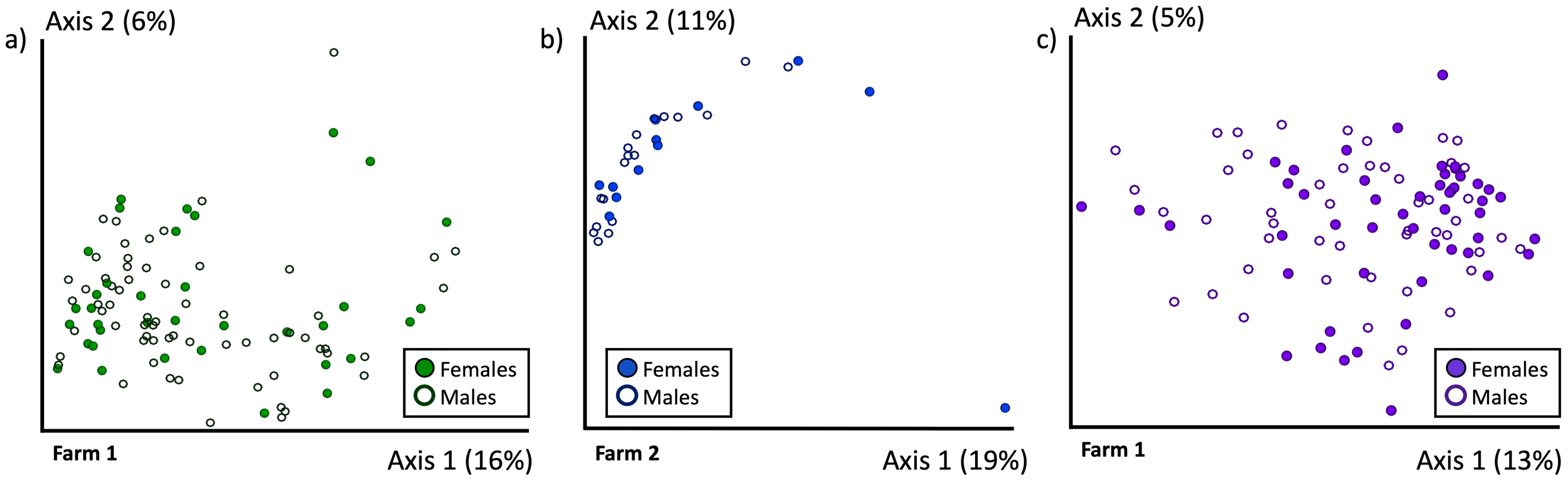
**

**Supplemental Figure 3 – Microbial Composition by CWD Status**

Gut microbial composition (unweighted UniFrac) by CWD Status on a) Farm 1 and b) Farm 2. Gut microbiota differed significantly by sex on Farm 1 (PERMANOVA p = 0.003), and on Farm 2 (PERMANOVA p = 0.003).

**
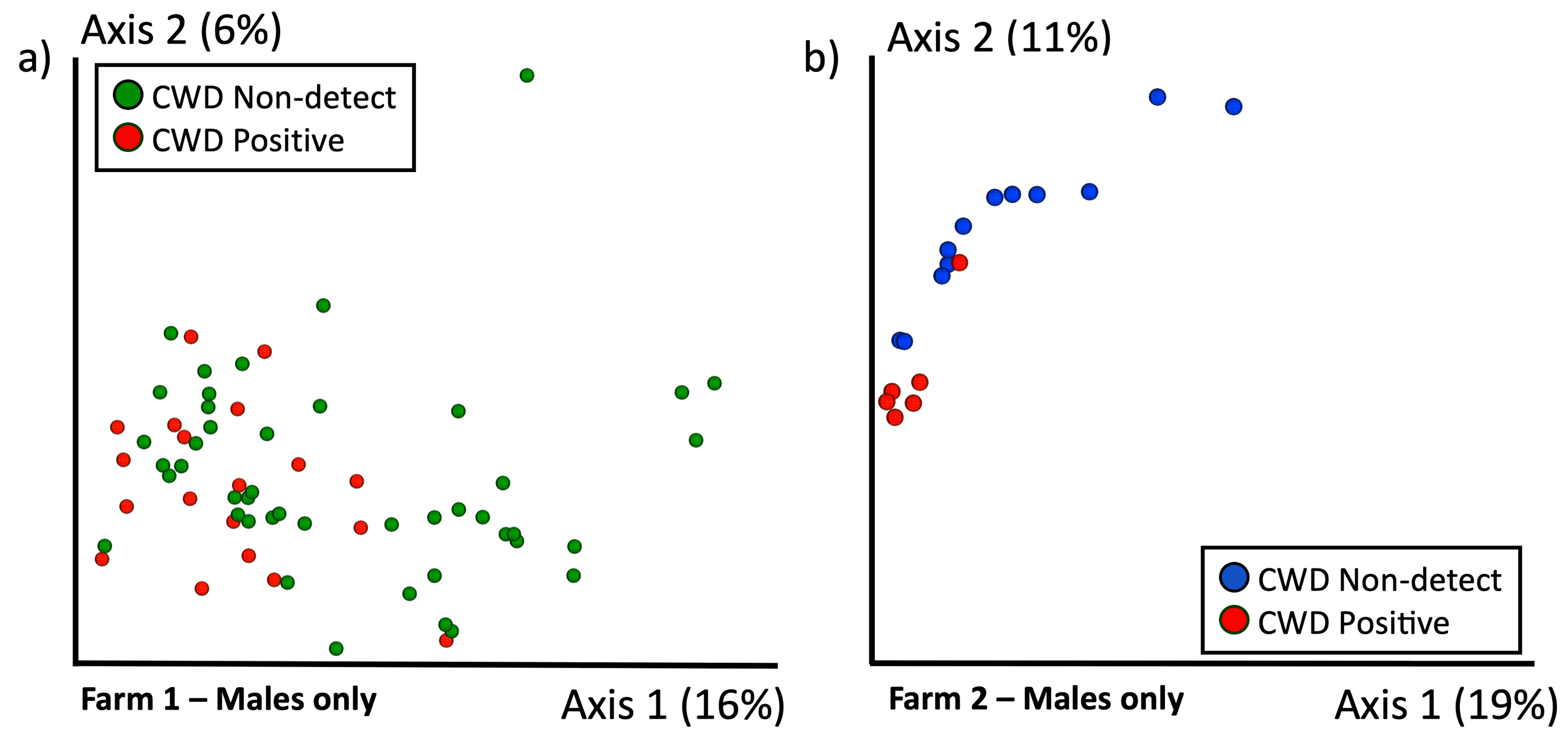
**
